# An updated meta-analysis of device related thrombus following left atrial appendage closure in patients with atrial fibrillation

**DOI:** 10.3389/fcvm.2022.1088782

**Published:** 2022-12-23

**Authors:** Song Zhang, Si-huai Xiong, Yu-gen Guan, Xian-xian Zhao, Yong-wen Qin, Zhi-fu Guo, Yuan Bai

**Affiliations:** Department of Cardiovasology, Shanghai Changhai Hospital, Naval Medical University, Shanghai, China

**Keywords:** atrial fibrillation, left atrial appendage closure, stroke, stroke prevention, thromboembolism

## Abstract

**Aims:**

Device related thrombus (DRT) is a known complication of left atrial appendage closure (LAAC). However, the relation between DRT and elevated risk of ischemic events remains controversial. This study is sought to reassessed the incidence of DRT following LAAC and the relation between DRT and elevated risk of ischemic stroke and systemic embolism (SE) with latest clinical trials included.

**Methods:**

The PubMed, Embase, and Cochrane Library databases were systematically searched from their inception until April 2022 for studies that reported the incidence of DRT and compared the incidence of both stroke and SE between DRT patients and non-DRT patients.

**Results:**

In 59 eligible studies, the incidence of DRT was 366/12,845 (2.8%, ranging from 0 to 11%, *I*^2^ = 64%). The incidence of DRT was not statistically different between single-seal device (SS) and dual-seal device (DS) in subgroup analysis [171/6,190 (2.8%) vs. 78/3,023 (3.6%); *p* = 0.93]. The pooled incidence of stroke (26 studies, 7,827 patients) in patients with and without DRT was 11.5% in DRT patients and 2.9% among non-DRT patients (OR: 5.08; 95% CI = 3.47–7.44). In the sensitivity analysis, DRT was associated with higher rate of stroke (12.1 vs. 3.2%; OR: 4.14; 95% CI = 2.69–6.38) and SE (16.0 vs. 3.8%; OR: 4.48; 95% CI = 3.04–6.62).

**Conclusion:**

The incidence of DRT was low and similar between SS and DS devices. DRT was associated with increased rates of ischemic events. The occurrence rate of ischemic events associated DRT was comparable between two occlusion mechanism devices.

**Systematic review registration:**

[https://www.crd.york.ac.uk/], identifier [CRD42022326179].

## Introduction

Atrial fibrillation (AF) is one of the most common arrhythmias in clinical practice among the elderly (1). Moreover, AF is related to increased risk of several serious adverse events such as ischemic stroke and systemic embolism (SE) (2). Research have shown that patients with AF are four to five times more likely to develop thrombotic and embolic events than the general population (3, 4). Therefore, stroke prevention with oral anticoagulation (OAC) and direct oral anticoagulants (DOACs) is an important part of the treatment regime in patients with AF (5). However, it might be difficult to use these drugs in the clinic, for drug interactions may increase the bleeding risk in patients. Due to these limitations, left atrial appendage closure (LAAC) is recommended for patients who are intolerant to OAC (6). Growing operator experience coupled with technical improvements and device modification have reduced procedural complications and accelerated continued growth in LAAC (7). However, device related thrombus (DRT) is still a known complication of LAAC. Until now, the prevalence and possible risk factors of DRT were discussed based on the randomized trials and large registries (8–11). Saw et al. (9) concluded that independent predictors of DRT were smoking and female sex, while Plicht et al. (10) thought CHADS_2_ and CHA_2_DS_2_-VASc scores, platelet count, and ejection fraction were risk factors of DRT formation. Nevertheless, whether DRT increases the risk of stroke and SE remains controversial. In Cochet’s study (12), a non-significant trend for a lower rate of stroke and SE was observed in DRT group rather than normal patients; while in a large multicenter RCT led by Dukkipati et al. (13) concluded that a non-significant trend for higher occurrence rate of stroke and SE was observed in DRT group. It has been less clear if and to what degree the discovery of this finding increases the stroke risk (14). Recently, major published studies focused on the incidence and consequence of DRT, hence we conducted an updated meta-analysis of both latest observational cohort and randomized controlled trials (RCT) to investigate the association of DRT following LAAC with stroke and SE. Furthermore, the subgroup analysis was performed to explore whether there was a causal relationship between DRT and device types and post-implant anti-thrombotic strategy.

## Materials and methods

### Search strategy

We conducted a systematic search for relevant articles in PubMed, Embase, and Cochrane Library up to April 2022. The following keywords were used: “Left Atrial Appendage Closure/Occlusion,” “device thrombosis/thrombus/embolization,” “device related thrombus/ thrombosis,” and “device associated thrombus/thrombosis.” Both mesh terms and free terms were used to search for relevant studies. In addition, we searched the references of identified studies to find other satisfactory studies. Our meta-analysis was conducted and reported according to the Preferred Reporting Items for Systematic Reviews and Meta-Analyses Protocols (PRISRMA-P) (15). This task was completed by two reviewers (SZ and S-HX) independently. The full text of relevant papers will be reviewed if the titles and abstracts of the articles cannot provide enough information for our inclusion. When disagreements arose, a third reviewer (X-XZ or Z-FG) was consulted. The initial study protocol was pre-registered at PROSPERO (CRD42022326179).

### Inclusion and exclusion criteria

The inclusion criteria were as following: (1) Patients received LAAC with left atrial occlusion device (Watchman, ACP, or Amulet); (2) With clearly outcomes of our interest reported. The outcomes were DRT, stroke, and SE. The definition of these outcomes and the classification of devices of LAAC (16) was showed in Table 1. Specifically, the single-seal (SS) device includes Watchman, Watchman FLX, Occlutect device, and the dual-seal (DS) device includes ACP, Amulet, LAmbre device; (3) The patients were followed-up for at least 6 months; (4) At least 30 patients recruited in the study; (5) Studies were published in English.

**TABLE 1:** Definition of device related thrombus (DRT), ischemic events, and classification of devices.

Terms	Definition
DRT	A well-circumscribed echoreflective mass by *trans*-esophageal echocardiographic imaging on the left atrial side of the device (during 3–12 months) (48).
Stroke	Ischemic stroke, the time of occurrence is at least 6 months after LAAC.
SE	Including ischemic stroke, TIA, and peripheral embolism (48).
Single-seal device (SS)	Including Watchman, Watchman FLX, Occlutect device.
Dual-seal device (DS)	Including ACP, Amulet, LAmbre device.

DRT, device related thrombus; SE, systemic embolism.

The exclusion criteria were as following: (1) Studies which were not belong to clinical trials; (2) Studies included other interventions excluded LAAC; (3) Data from non-human species; (4) Duplicate reports with identical data; (5) The imaging detection of DRT should be performed at least 3 months after LAAC.

### Quality assessment

Cochrane Collaboration risk of bias (Cochrane ROB) (17) instrument was used to perform quality assessment for the RCTs. All the observational studies were assessed using the adapted methodological items for non-randomized studies (MINORS) (18). Strictly according to the scoring criteria, two reviewers (SZ and S-HX) assessed the quality of two types of studies independently. A third reviewer (X-XZ or Z-FG) was consulted when disagreements arose.

### Extraction

The data were extracted from the selected studies by two researchers (SZ and S-HX) independently. When disagreements arose, a third researcher (YB or Y-WQ) was consulted. The extracted data were as follows: first author’s name, basic characteristics, LAAC device, the type of anti-thrombotic therapy, the follow-up date and protocol and the rate and diagnosis time of DRT, stroke and SE. In addition, in case of multiple studies from the same pool of patients, we will include the most recent study only (19).

### Statistical analysis

We combined of each article using standard meta-analytic methods to estimate overall incidence of DRT and compared the incidence of both stroke and SE in DRT patients with non-DRT patients (defined as patients received LAAC without the occurrence of DRT). Revman (Version 5.4, The Cochrane Collaboration, London, UK) and Statistical Product and Service Solutions (SPSS) were used to analyze available data. For the incidence of DRT, stroke and SE each eligible studies, pooled estimates of odds ratio with 95% confidence interval (CI) were calculated (20, 21). The mean difference (MD) and 95% CI were used to estimate the basic characteristics of patients. Chi-square and *I*-square tests were used for statistical heterogeneity measurement between eligible studies. A *p*-value < 0.05 was considered to be statistically significant. A fixed-effects model was used if significant heterogeneity (*I*^2^ < 50%) was found among those studies. Otherwise, a random-effect model was used (22). Sensitivity analysis was performed to investigate potential source of inconsistency by including multicenter registries (MCR) and randomized controlled studies (RCT) only. Funnel plot and Begg’s test were used to access publication bias (Supplementary Figure 1). Furthermore, to find out the potential predictor of DRT, eligible studies with DRT predictors [effect estimate with risk ratio (RR)] were included in secondary analysis. Separate meta-analyses were conducted for predictor variables that were reported in at least three different studies (23). When two or more RR were present per predictor, the pooled RRs with 95% CI were computed using random model (24).

## Results

### Search results and quality assessment

**FIGURE 1:**
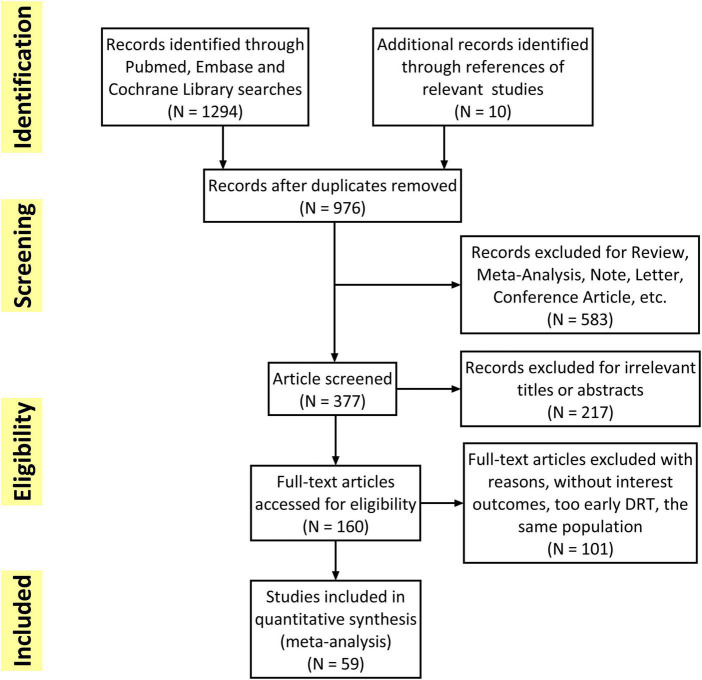
Flowchart of eligible studies.

The flowchart of the study selection was presented in Figure 1. Overall, 1,304 studies were initially retrieved and a total of 59 studies met the inclusion criteria and were finally included. Subsequently, 101 studies were excluded because of without interest outcomes (DRT, stroke or SE), too early DRT (detection in less than 3 months) and unacceptable time for imaging follow-up. The quality of all included studies was shown in Supplementary Table 4. The mean MINORS score for the observational studies was 10.7 ± 1.50. The only one RCT (25) assessed by Cochrane ROB showed low risk in all eight items.

### Study characteristics

All the included studies were clinical trials with 6 months to several years follow-up. The eligible studies enrolled 12,845 patients with sample size ranging from 30 to 1,739. Most of the patients were elderly people (73.48 ± 8.64 years) and 60% of them had permanent AF. The mean CHA_2_DS_2_–VASc score, and HAS-BLED score was 4.11 ± 1.50 and 2.94 ± 1.19, respectively. About the device used in appendage occlusion, 60% patients used the SS device while the remaining 40% used DS device. The total procedural success rate among all the patients was 95%. Antithrombotic therapy at discharge was OAC in 19%, DOAC in 5%, single/dual antiplatelet (SAPT/DAPT) in 48%, and other/not reported (NR) in 28%. The pooled baseline data of patients in the included studies was summarized in Table 2. The detailed data of each eligible studies was summarized in Supplementary Table 1.

**TABLE 2:** Baseline characteristics of the studied patients (*N* = 12,845).

Age	73.48 ± 8.64
**Type of atrial fibrillation**	
Paroxysmal	40
Permanent	60
Hypertension	83
Diabetes mellitus	29
Previous stroke/transient ischemic attack	34
Peripheral vascular disease	22
Carotid disease	10
Coronary disease	40
Congestive heart failure	22
Chronic renal insufficiency	13
Mean CHA_2_DS_2_–VASc score	4.11 ± 1.50
Mean HAS-BLED score	2.94 ± 1.19
**Appendage occlusion device used**	
Single-seal device (SS)	60
Dual-seal device (DS)	40
Procedural success	95
**Antithrombotic therapy at discharge**	
OAC	19
DOAC	5
N/S (OAC/DOAC)	8
DA	39
SA	9
N/R	20
Number of the patients with follow-up imaging	12,468 (97)
The number of DRT	366/12,845 (2.8)
The number of late DRT (>365 days)	22
**Ischemic events in the follow-up**	
Stroke	314
Systemic embolism	439
**DRT treatment**	
LMWH	4
OAC	22
SAPT	5
DAPT	8
N/R	61

Values are mean ± SD, %, *n* (%), or *n/N* (%). OAC, oral anticoagulation; DOAC, direct oral anticoagulants; DAPT, dual antiplatelet; LMWH, low-molecular-weight heparin; DRT, device related thrombus.

### DRT and ischemic events

**FIGURE 2:**
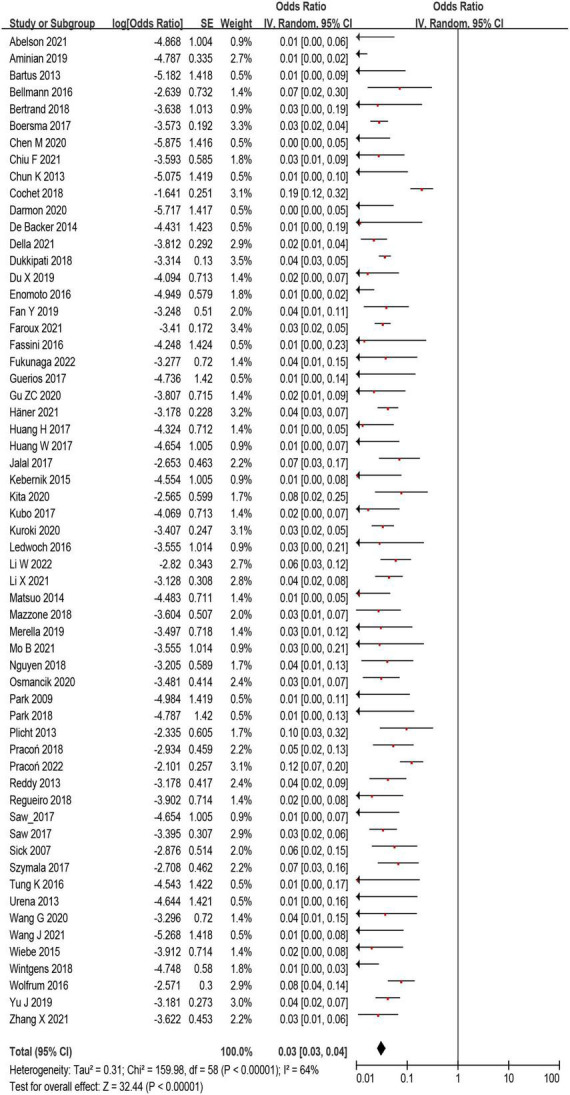
The forest plot of the device related thrombus (DRT) incidence after left atrial appendage closure (LAAC) of all eligible studies. The odds ratio represents the incidence of DRT.

**FIGURE 3:**
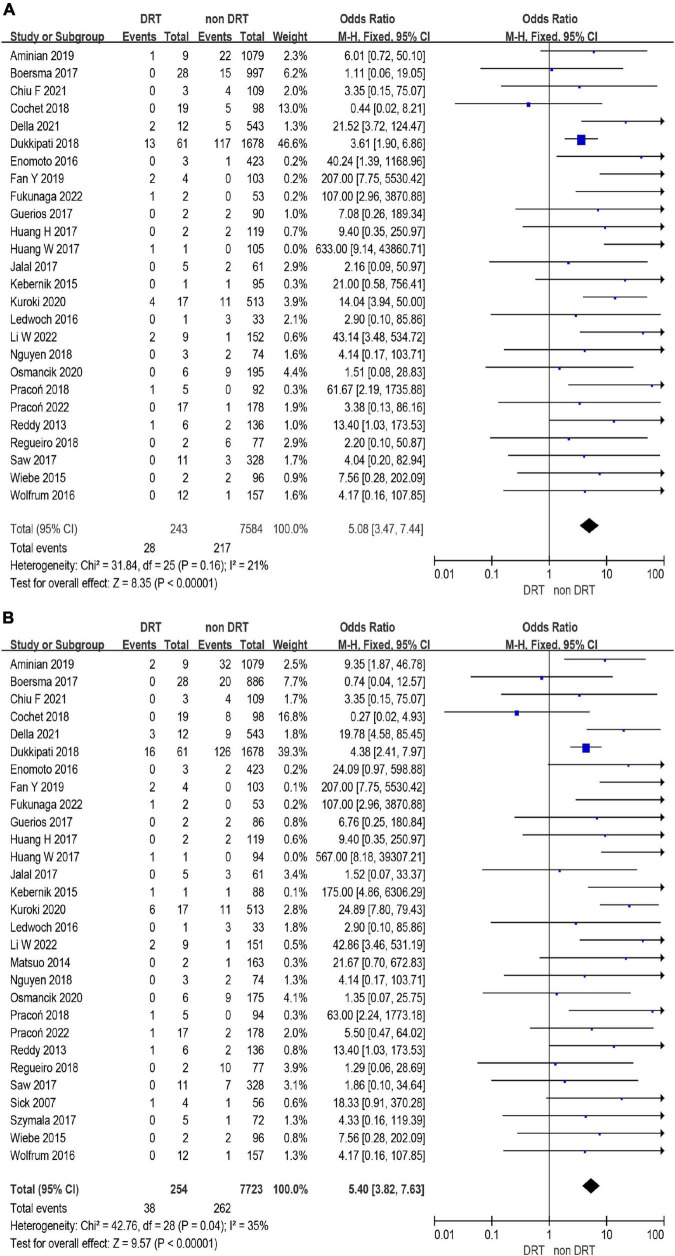
Forest plots of stroke **(A)** and systemic embolism (SE) **(B)** in patients with device related thrombus (DRT) and non-DRT.

A total of 12,468 (97%) patients had at least once imaging follow-up for LACC device. The number of DRT, and late DRT was 366 and 22, respectively. Among all patients included in our study, the pooled incidence of DRT was 2.8 (366/12,845), with significant variation in the reported incidence (ranging from 0 to 11.3%; *I*^2^ = 64%) (Figure 2). In the follow-up period, 314 ischemic stroke events and 439 SE were detected. Studies which reported the specific number of ischemic events between DRT patients and non-DRT patients were included for additional analysis. In the studies that reported the incidence of stroke between DRT and non-DRT (26 studies, 7,827 patients), the pooled incidence of stroke was 11.5% in DRT patients and 2.9% among non-DRT patients (OR: 5.08; 95% CI = 3.47–7.44; *p* < 0.001; *I*^2^ = 21%) (Figure 3A). And in the studies that reported the incidence of SE (29 studies, 7,977 patients), the pooled incidence of SE was 15.0% and 3.4% among patients without DRT (OR: 5.40; 95% CI = 3.82–7.63; *p* < 0.001; *I*^2^ = 35%) (Figure 3B).

### Sensitivity analysis

In a sensitivity analysis, we conducted a meta-analysis to compare the incidence of ischemic events between DRT patients and non-DRT patients in MCR and RCT only. In these studies (25 studies, 9,320 patients), the pooled incidence of DRT was 2.8% (259 of 9,320) (Supplementary Figure 2). In the comparison of ischemic events in these studies, the DRT was associated with higher rate of stroke (12.1 vs. 3.2%; OR: 4.14; 95% CI = 2.69–6.38; *p* < 0.001; *I*^2^ = 24%) and SE (16.0 vs. 3.8%; OR: 4.48; 95% CI = 3.04–6.62; *p* < 0.001; *I*^2^ = 44%) (Supplementary Figures 3, 4).

### Subgroup analysis

**FIGURE 4:**
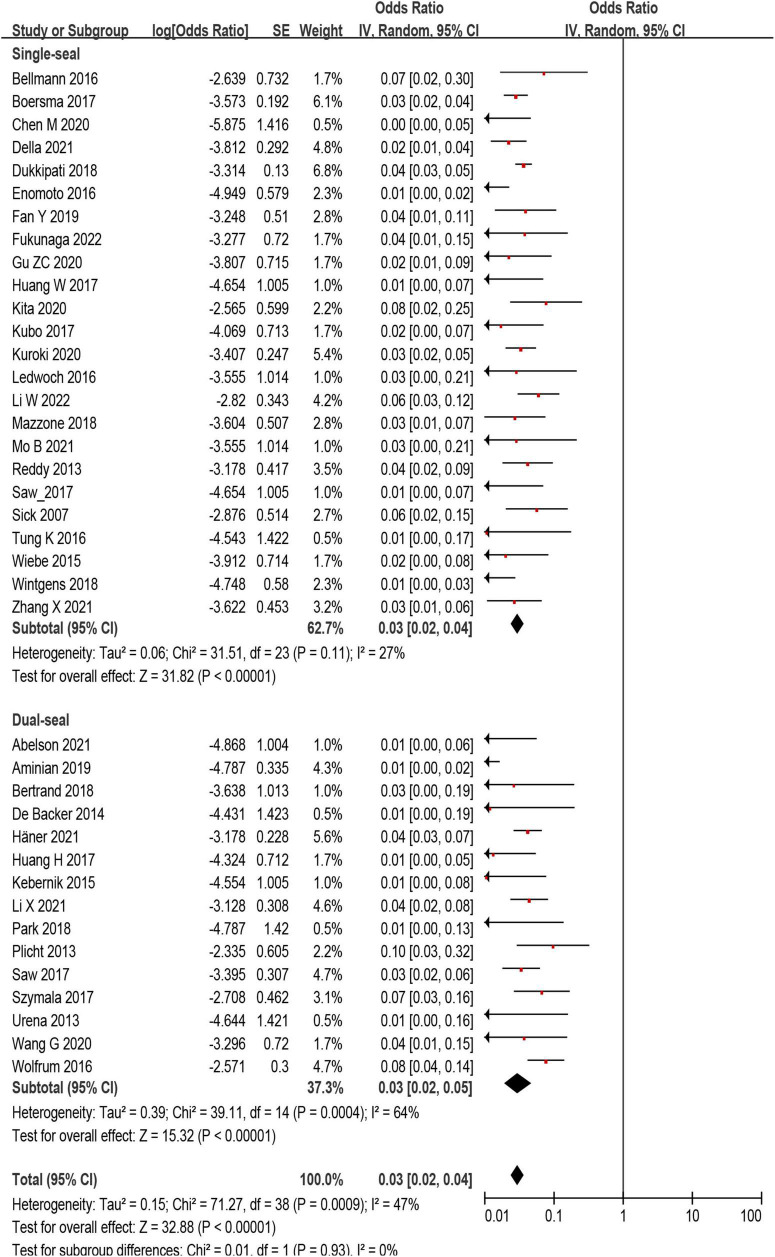
Forest plot comparing the incidence of device related thrombus (DRT) with subgroup analysis based on occlusion mechanism.

**FIGURE 5:**
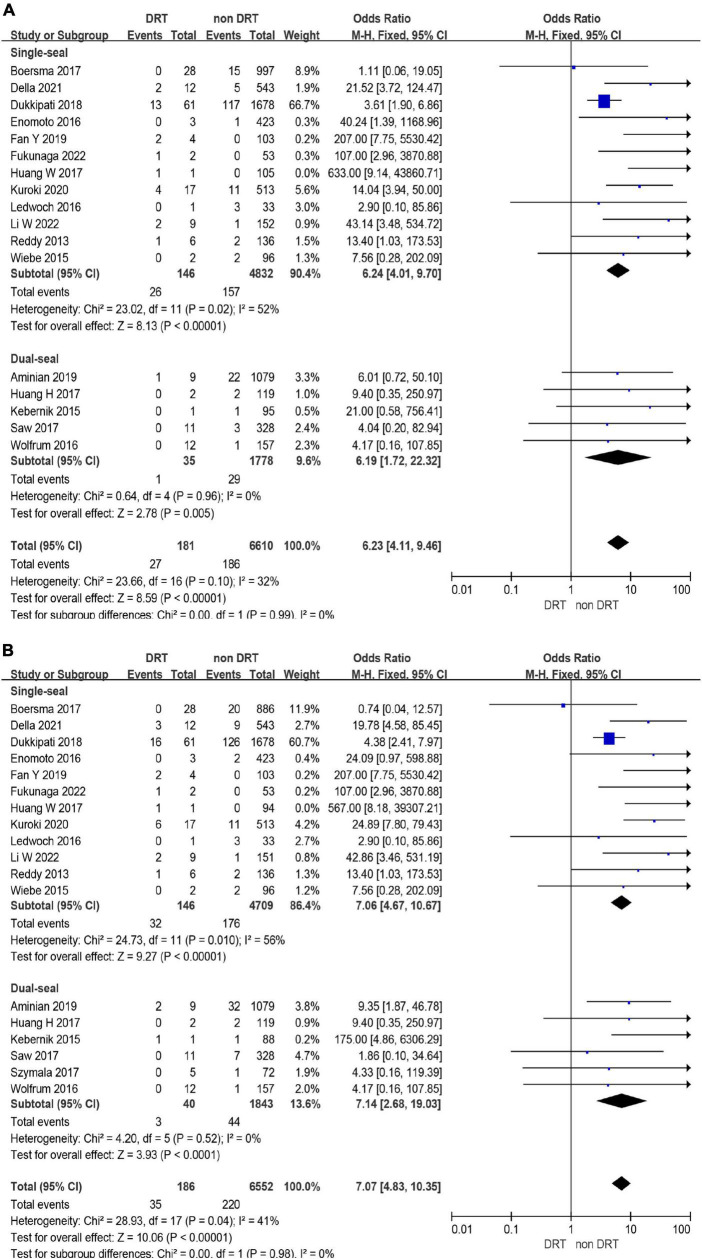
Forest plots of stroke **(A)** and systemic embolism (SE) **(B)** in device related thrombus (DRT) and non-DRT patients with subgroup analysis based on occlusion mechanism.

The incidence of DRT was not statistically different in patients who underwent LAAC using SS vs. DS device [171/6,190 (2.8%) vs. 78/3,023 (3.6%); *p* = 0.93] (Supplementary Tables 2, 3 and Figure 4). In the subgroup analysis, the incidence of stroke (*p* = 0.99) and SE (*p* = 0.98) associated DRT was not statistically different between SS and DS devices (Figure 5). And in the subgroup analysis based on anti-thrombotic therapy, the difference of the incidence of DRT was similar in patients who received OAC vs. APT (*p* = 0.21) (Supplementary Figure 5).

### Resolution of DRT

Among all the patients diagnosed with DRT, 144 patients (39%) were reported using specific regimen to treat DRT. In these patients, 16 patients (4%) received low-molecular-weight heparin (LMWH), 81 patients (22%) received OAC, and 47 patients (13%) were treated as SAPT or DAPT. In the follow-up period, most of the DRT was resolved by drugs, while only few patients (<5%) needed surgical intervention.

### Predictive and non-predictive factors of DRT

**FIGURE 6:**
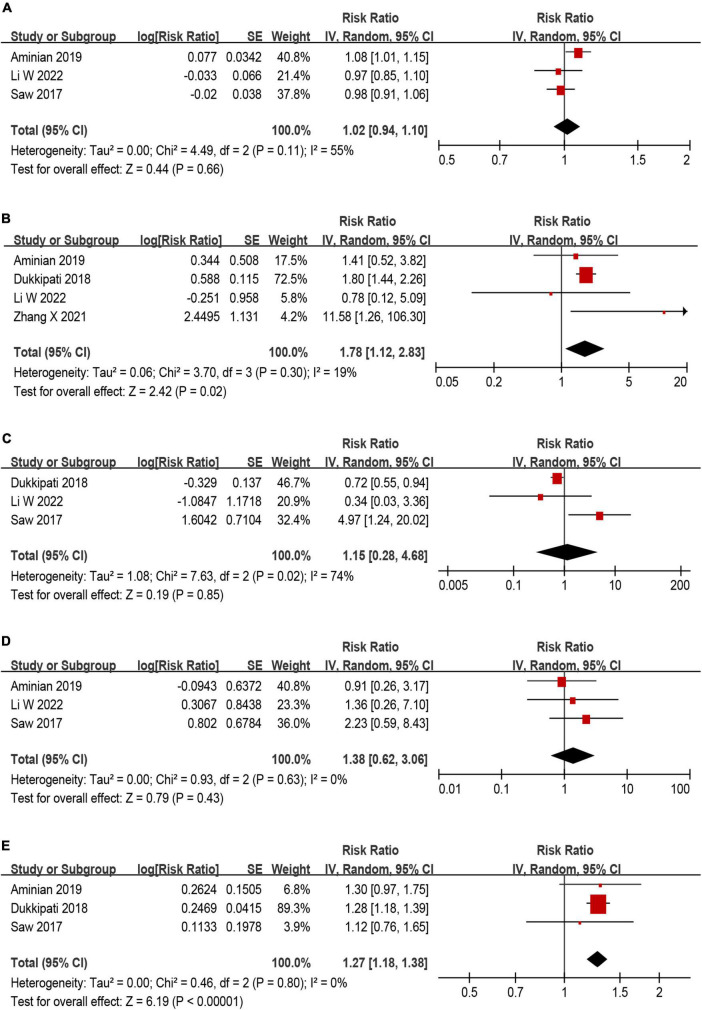
Forest plot of risk ratios (RR) for **(A)** age, **(B)** history of stroke/TIA, **(C)** smoking, **(D)** hypertension, and **(E)** CHA_2_DS_2_–VASc score as predictors for device related thrombus (DRT).

Among those eligible studies, five studies (9, 13, 26–28) investigating potential predictors of DRT were included. Five predictors were used in the meta-analysis, including age, smoking, history of stroke/TIA, hypertension and CHA_2_DS_2_–VASc score (Figure 6). Of the five predictors included in the current meta-analysis, history of stroke/TIA and CHA_2_DS_2_–VASc score remained statistically significant (Figures 6B, E), while the other three predictors (age, smoking, and hypertension) were non-predictive after effect size aggregation (Figures 6A, C, D). Only the meta-analysis of smoking predictor showed high heterogeneity.

## Discussion

We performed a systematic review to assess the incidence of DRT and whether detected DRT is associated with a significantly elevated risk of ischemic events. The clinical trials in our meta-analysis included more than 12,800 patients (largest-to-date) and have established a solid evidence base supports that DRT is associated with a fivefold increase in ischemic events. The incidence of DRT after LAAC in included studies was 2.8%, which is lower than previous meta-analysis describing the occurrence rate of DRT (9). In the subgroup analysis of anti-thrombotic therapy, the incidence of DRT was similar between patients received post-implant APT or OAC. While the subgroup analyses based on occlusion mechanism showed the incidence of DRT was not statistically different in patients who underwent LAAC using DS or SS device. And the elevated risk of ischemic events associated DRT was not related to occlusion mechanisms of the device.

On the basis of the early successes of PROTECT-AF and PREVAIL trial, LAAC became rapidly adopted as stroke preventive strategy (20). Several major complications have been reported including pericardial tamponade and device embolization (29). However, DRT remains a major concern, with an estimated incidence of 4% (range 0–16% in eligible studies) (12, 30). In previous studies, plenty studies set 45 days after LAAC for DRT detection (31–35). However, DRT requires a certain development time after device implantation. Therefore, in combination with the definition of DRT in multiple literatures, the occurrence time of DRT was defined as at least 75 days after LAAC in our study. Studies which DRT reported within 3 months after LAAC were not included. Additionally, our meta-analysis included 59 eligible studies enrolled 12,845 patients with sample size ranging from 30 to 1,739. Compared with the previous meta-analysis, our study has included more literature published in the past 3 years. Also, more strictive definition of occurrence time was used in our study to calculate the pooled incidence of DRT. And this increased the credibility of our DRT incidence results. Therefore, the major procedure-related complication incidence has improved over time and with increasing physician. Furtherly, improvements can be made to the current device to further reduce the incidence of DRT (WATCHMAN FLX vs. WATCHMAN 2.5; Amulet vs. ACP) (36).

Current published reports suggests that DRT is associated with a significantly elevated risk of ischemic events (9, 13, 26). A significant positive association between the incidence of DRT and subsequent ischemic events was also observed in our meta-analysis, documented approximately fivefold greater rates of ischemic events in patients with DRT compared with those without DRT. Among patients who developed DRT and stroke, majority of them were diagnosed with DRT prior to the occurrence of stroke. Although different clinicians did not have a uniform opinion on the relationship between DRT and stroke, the persistent signal of increased ischemic events warrants attention. However, current result together indicate that DRT after LAAC represents a significant danger in patients with AF who are already at high risk for stroke or TIA. Ding et al. (37) suggested that the high-risk AF patients have a high rate of mortality and an ongoing significant risk of bleeding and thrombotic events during follow-up. While Teiger et al. (38) concluded that LAAC in high-risk patients seems reasonable to decrease the rate of stroke. The limitations of the uncertain definition of DRT occurrence time reduce the credibility of their results. It is noteworthy that the thrombus development after LAAC is not clear since DRT is mostly silent and its follow-up is highly depended on imaging tools. As a result, it is difficult for clinicians to diagnose the DRT occurrence time precisely. It is important to detect them in a timely manner during follow up through appropriate diagnostic imaging techniques such as coronary angiography and *trans*-esophageal echocardiography. Our findings suggest that DRT is associated with more severe complications (stroke and SE), so early diagnosis and treatment of DRT may lead to better prognosis of patients. However, some studies have shown that anticoagulant therapy has an effect on the endothelialization process (39–41), and the occurrence of DRT is related to the incomplete endothelialization of device, so the early use of anticoagulant to treat DRT may hinder the completion of endothelialization. Recent studies indicated a reduced risk of DRT in patients receiving post-LAAC anticoagulation (42).

Within the last decade, several LAAC devices have been developed and introduced into clinical practice. The included clinical trials in our study also covered 3–4 types of devices. In our study, the devices are divided into two categories, one is SS including Watchman, FLX, Occlutect, and the other is DS including ACP, Amulet, LAmbre (16). The subgroup analyses showed that the incidence of DRT was not statistically different between SS and DS group, and the occurrence rate of stroke and SE associated DRT was similar between two groups. Based on these results, it seemed that the safety for LAAC was comparable between devices under different occlusion mechanisms. As more clinical trials comparing the clinical outcomes between SS and DS devices are underway, it is important to consider not only the baseline characteristics of the patient but also the differences in device mechanisms when formulating post-operative therapy.

Identifying predictors or risk factors of DRT is important for DRT prevention. As mentioned in the preceding text, the independent predictors of DRT occurrence remained unknown. In our current meta-analysis, the predictors of DRT were history of stroke/TIA and CHA_2_DS_2_–VASc score, while the conclusion among studies were inconsistent. Saw et al. (9) concluded that smoking and female sex were independent predictors of DRT, while Plicht et al. (10) suggested that predictors might be CHADS_2_ and CHA_2_DS_2_–VASc scores, platelet count, etc. In our previous real-world study, peri-device leak was considered as a risk factor of DRT (43). In recent two studies deep implantation depth was found to be a risk factor for DRT (7, 42). Though other predictors such as pericardial effusion and renal insufficiency were found, these predictors should be confirmed in larger studies (7). Until more data become available, researchers should raise more concern on the treatment of DRT, though the selection of treatment is still in debate. Simard et al. (44) concluded that 8–12 weeks Vitamin K antagonist (VKA), NOAC, and 2–4 weeks LMWH were recommended for treatment of DRT. Sedaghat et al. (45) revealed that patients underwent the three regimens mentioned above showed no statistical difference in clinical outcomes. Patients diagnosed with DRT may have persistent DRT or DRT recurrence after receiving anti-thrombotic therapies. Recent studies reported that only approximate a quarter of cases demonstrating persistent DRT presence in clinical follow-up (7, 45). And patients with persistent DRT showed higher stroke rates and increased mortality rates (45). And Asmarats et al. (46) firstly evaluated the recurrence of DRT, suggesting that thrombus recurrence was common, thus long-term OAC was encouraged after a first DRT. More specified anti-thrombotic regimens and more RCT are needed in prevention of DRT. Except for DRT, late DRT is thought to be less frequent because of device sealing, which remains variable on an individual basis. Furthermore, the collection of late DRT in this study is not comprehensive enough to carry out effective analysis. Consistent with our results, Sedaghat et al. (45) suggested that no clinical or echocardiographic predictors for late DRT formation could be identified in their study.

This meta-analysis has several limitations. First, because of limited randomized data, this meta-analysis included both randomized and observational studies. The observational studies are subjected to unmeasured confounding and selection bias. Therefore, consistent with previous meta-analysis of DRT (47), we conducted sensitivity analysis by including MCR and RCT only to test the inconsistency, which could verify the feasibility of the inclusion of multiple studies. Second, the definition of some clinical events such as major procedure-related complications was not unanimous across studies; however, it was less likely to have a huge impact on our final conclusion. Third, the follow-up duration in each study varied, which may have negative influence on outcomes. Last but not least, due to the paucity of individual data in each eligible study, no subgroup analysis was done according to different generations of SS and DS.

## Conclusion

Device related thrombus is an infrequent complication of LAAC, associated with increased rates of ischemic events. The incidence of DRT was comparable between SS and DS devices. The occurrence rate of stroke and SE associated DRT was similar in devices with different occlusion mechanisms. Further large multicenter prospective studies are needed to confirm the true prevalence of DRT and to evaluate the risk factors, associated complications and treatment regimens.

## Data availability statement

The original contributions presented in this study are included in the article/Supplementary material, further inquiries can be directed to the corresponding author.

## Author contributions

SZ: data curation (lead), formal analysis (equal), investigation (equal), methodology (equal), software (lead), validation (equal), visualization (equal), writing – original draft (equal), and writing – review and editing (equal). S-HX: data curation (equal), formal analysis (equal), investigation (equal), methodology (equal), validation (equal), visualization (equal), writing – original draft (equal), and writing – review and editing (equal). Y-GG: methodology (equal), software (lead), and writing – review and editing (equal). X-XZ and Z-FG: supervision (equal), investigation (equal), methodology (equal), and writing – review and editing (equal). Y-WQ: supervision (equal), investigation (equal), and writing – review and editing (equal). YB: conceptualization (lead), resources (lead), writing – original draft (equal), writing – review and editing (equal), supervision (lead), and taken responsibility for all aspects of the reliability and freedom from bias of the data presented and their discussed interpretation. All authors contributed to the article and approved the submitted version.
