# Supplementary material for: An updated meta-analysis of device related thrombus following left atrial appendage closure in patients with atrial fibrillation
Source: Front Cardiovasc Med. 2022 Dec 23;9:1088782. doi: 10.3389/fcvm.2022.1088782 (PMC9816128; doi:10.3389/fcvm.2022.1088782)
Supplement: Supplementary file 1 [file Data_Sheet_1.docx]

**Supplementary Materials**

**Supplementary Table 1.** Individual Study Data of the Eligible Study included in the meta-analysis.

**Supplementary Table 2.** Comparisons of the incidence of DRT and related ischemic events among Single-seal (SS), Dual-seal (DS) and other devices.

**Supplementary Table 3.** Comparison of the incidence of DRT and related ischemic events among different type of devices.

**Supplementary Table 4.** Methodological quality assessment of 59 studies.

**Supplementary Figure 1.** Funnel plot for publication bias in eligible studies.

**Supplementary Figure 2.** The incidence of DRT after LAAC in multicenter registries and RCT.

**Supplementary Figure 3.** Forest plot of stroke in DRT patients and non-DRT patients: MCR and RCT.

**Supplementary Figure 4:** Forest plot of systemic embolism in DRT patients and non-DRT patients: MCR and RCT.

**Supplementary Figure 5.** Forest plot of incidence of DRT in patients with subgroup analysis based on anti-thrombotic therapy.

**Supplementary Table 1.** Individual Study Data of the Eligible Study included in the meta-analysis.

| **Study Name /Publication Year** | **Type** | **N** | **Age(mean ± SD)** | **CHA_2_D-VASC_2_** | **HAS-BLED** | **LAAC Device** | **OAC/DAPT** | **F/u** | **F/u LAA** | **DRT (%)** | **DRT** | **Stroke (%)** | **SE (%)** |
| --- | --- | --- | --- | --- | --- | --- | --- | --- | --- | --- | --- | --- | --- |
|  |  |  |  |  |  |  | **At d/c** |  | **Imaging Protocol (day)** |  | **Dx Time (Days)**  **n** |  |  |
| Abelson 2021 | SCR | 131 | 74.2 ± 8.1 | 3.9 ± 1.2 | 3.0 ± 0.5 | A | 4%O, 96%D | 114 | TTE at 30,180, 360 | 0.8 | N/R | 1.8 | 2.6 |
| Aminian 2019 | MCR | 1088 | 75.2 ± 8.5 | 4.2 ± 1.6 | 3.3 ± 1.1 | A | 42%O, 58%D | 1088 | TEE at 30, 90; CT-variable | 0.8 | 4(90), 3(180), 2(210day) | 2.4 | 3.1 |
| Bartus 2013 | SCR | 89 | 62 ± 10 | 2.8±1.6 | 2.4±1.2 | Lariat | N/R | 85 | TEE at 30, 90 | 0 | - | 0 | 1.1 |
| Bellmann 2016 | SCR | 30 | 74 ± 8 | 4 ± 1 | 4 ± 1 | Occlutech | N/R | 28 | TEE at 30, 90, 365 | 7.1 | 2(90 day) | 0 | 1 |
| Bertrand 2018 | SCR | 39 | 77 ± 7 | 5 (3-6) | 3 (3-4) | A | 100%D | 39 | TEE at 30, 60, 135 | 2.6 | 1(135 day) | 0 | 0 |
| Boersma 2017 | MCR | 1025 | 73.4 ± 8.9 | 4.5±1.6 | 2.3±1.2 | W | 27%O, 60%D | 914 | TEE – variable (75-225) | 3.1 | 28(<180 day) | 1.6 | 2.2 |
| Chen M 2020 | SCR | 178 | 68.9 ± 8.1 | 3.3 ± 1.5 | 1.6 ± 1.0 | W | N/R | 178 | TTE/TEE/CTA at 90 | 0 | - | 0.6 | 0.6 |
| Chiu F 2021 | MCR | 112 | 71.9 ± 10.0 | 4.1 ± 1.6 | 3.4 ± 1.8 | 50%A, 50%W | N/R | 112 | TEE at 60-120, 365 | 2.7 | N/R | 3.6 | 3.6 |
| Chun K 2013 | SCR | 80 | 76 ± 9 | 4.3 ± 1.7 | 3.1 ± 1.1 | 50%A, 50%W | 24%O, 76%D | 78 | TEE at 45, 90 | 0 | - | 0 | 0 |
| Cochet 2018 | MCR | 117 | 74 ± 9 | 4.4 ± 1.3 | 3.5 ± 1.0 | 71%A, 29%W | N/R | 117 | CTA at 90 | 16 | 19(90 day) | 4.3 | 6.8 |
| Darmon 2020 | SCR | 152 | 79.3 ± 7.2 | 4.4 ± 1.1 | 3.8 ± 1.1 | 81%A, 19%W | 1%O, 99%D | 152 | CTA at 45, 90 | 0 | - | 3.6 | 3.6 |
| De Backer 2014 | SCR | 42 | 74.6 ± 8.2 | 4.5 ± 1.4 | 3.7 ± 0.9 | A | 85%O, 15%D | 41 | TOE at 45-90 | 0 | - | 2.3 | 2.3 |
| Della 2021 | MCR | 555 | 75.1 ± 8.1 | 4 (3-6) | 3 (2-4) | W | 100%D | 555 | TEE at 45, 180 | 2.1 | 12(7.7 ± 4.2 month) | 1.3 | 0.7 |
| Du X 2019 | MCR | 122 | 66.4 ± 8.8 | 4.3 ± 1.4 | 3.3 ± 1.0 | 73%W, 27%A | 48%O, 52%D | 122 | TEE at 45, 180 | 1.6 | 2(180 day) | 0 | 0 |
| Dukkipati 2018 | RCT | 1739 | 73.8 ± 5.9 | 3.9 ± 1.5 | 2.0 ± 1.0 | W | N/R | 1739 | TEE at 45, 180, 365 in the RCTs and at 45 and 365 in the CAR | 3.5 | 16(45), 18(180), 27(365 day) | 7.5 | 8.2 |
| Enomoto 2016 | MCR | 426 | 75.5 ± 8.0 | N/S | N/S | W | 50%O, 50%D | 426 | TEE/CTA at 30, 120 | 0.7 | 2(120 day) | 0.2 | 0.5 |
| Fan Y 2019 | MCR | 107 | 72 ± 8 | 4.1 ± 1.5 | N/R | W | 51%O, 32%D | 107 | TEE at 30, 90 | 3.7 | 4(55-112day) | 1.9 | 1.9 |
| Faroux 2021 | MCR | 1094 | 75.9 ± 8.4 | 4.5 ± 1.5 | 3.6 ± 1.1 | 23%W, 77%A | 21%O, 44%D | 1094 | TEE at 90 | 3.2 | 35(90 day) | 2.2 | 3.9 |
| Fassini 2016 | SCR | 35 | 72 ± 4 | 3 ± 1.5 | 3 ± 1.2 | 29%W,71%A | 100%O | 35 | TEE at 180, 365 | 0 | - | 0 | 0 |
| Fukunaga 2022 | SCR | 55 | 74.1 ± 7.8 | 4.6 ± 1.4 | 3.8 ± 0.9 | W | 49%O, 51%D | 55 | CTA at 45, 180, 240 | 3.6 | 1(180), 1(240day) | 1.8 | 1.8 |
| Gu ZC 2020 | SCR | 57 | 72 (65-76) | 5 (3-6) | 4 (3-5) | W | 100%O | 57 | TEE at 45, 180, 365 | 0 | - | 0 | 0 |
| Guerios 2017 | MCR | 92 | 73.1 ± 10.1 | 4.5 ± 1.5 | 3.6 ± 1.0 | 5%W, 95%A | 100%D | 88 | TEE – variable | 2.3 | 2(346 day) | 2.2 | 2.2 |
| Häner 2021 | MCR | 500 | 73.9 ± 10.0 | 4.3 ± 1.7 | 3.0 ± 1.1 | A | 100%D | 500 | TEE – variable | 4 | 20(90-180day) | 0.4 | 1.8 |
| Huang H 2017 | MCR | 153 | 69.3 ± 9.4 | 4.0 ± 1.7 | N/R | Lambre | 100%D | 121 | TEE at 90, 365 | 1.3 | 2(90 day) | 1.3 | 1.3 |
| Huang W 2017 | SCR | 106 | 64.2 ± 8.6 | 3.6 ± 1.6 | N/R | W | 100%O | 95 | TEE at 45 | 0.9 | 1(153 day) | 2.1 | 2.1 |
| Jalal 2017 | MCR | 76 | 73 ± 8 | 4.4 ± 1.3 | 3.4 ± 0.9 | 11%W, 89%A | 3%O, 0%D | 66 | CTA at 90 | 7.5 | 5(90 day) | 3 | 4.5 |
| Kebernik 2015 | SCR | 96 | 76 ± 7 | 4 (3–4.75) | 3 (3–4) | A | 100%D | 89 | CTA at 180 | 1 | 1(180 day) | 1 | 2.1 |
| Kita 2020 | SCR | 42 | 71.1 ± 8.5 | 3.3 ± 1.1 | 2.5 ± 1.4 | W | 100%O | 42 | TEE at 90, 180 | 7.1 | 3(90/180day) | 0 | 0 |
| Kubo 2017 | SCR | 119 | 75.8 ± 9.3 | 4.2 ± 1.8 | 2.9 ± 1.2 | W | 100%O | 117 | TEE at 45, 180, 365 | 1.7 | 1(180), 1(365 day) | 0 | 0 |
| Kuroki 2020 | MCR | 530 | 78.7 ± 7.9 | 4.5 ± 1.4 | 3.0 ± 1.0 | W | N/S | 530 | TEE/CTA at 120 | 3.2 | 11(120), 2(365day), 4(N/S) | 2.8 | 3.2 |
| Ledwoch 2016 | SCR | 36 | 72 ± 16 | 4.6 ± 1.6 | N/R | W | 100%O | 34 | TEE at 45, 180 | 3 | 1(180 day) | 8.3 | 8.3 |
| Li W 2022 | SCR | 160 | 69.1 ± 6.1 | 3.7 ± 1.1 | 4.2 ± 0.8 | W | 51%O, 0%D | 160 | TEE/CTA at 90 | 5.6 | 9(90 day) | 1.9 | 1.9 |
| Li X 2021 | SCR | 262 | 69.7 ± 8.5 | 3.4 ± 1.5 | 3.1 ± 1.4 | A | 100%O | 250 | TEE at 90, 180, 365 | 4.4 | 11(90 day) | 4 | 7.6 |
| Matsuo 2014 | SCR | 179 | 72.7 ± 9.0 | 4.3 ± 1.5 | 3.9 ± 1.1 | 96%W, 4%A | N/R | 165 | TEE at 45, 180 | 1.1 | 2(180 day) | 0 | 0.6 |
| Mazzone 2018 | MCR | 151 | 73 ± 8 | 3.7 ± 1.3 | 3.3 ± 1.1 | W | 26%O, 0%D | 150 | TEE at 30, 180, 365 | 1.7 | 4(180 day) | 2 | 3.3 |
| Merella 2019 | SCR | 68 | 73.6 ± 8.7 | 3.7 ± 1.4 | 3.2 ± 1.0 | 12%A, 88%W | 3%O, 47%D | 62 | TEE at 90, 365 | 2.9 | 2(N/S) | 0 | 0 |
| Mo B 2021 | SCR | 36 | 69.1 ± 7.8 | 3.6 ± 2.0 | 2.0 ± 1.1 | W | 100%O | 36 | TEE at 45, 180 | 2.8 | 1(180 day) | 0 | 0 |
| Nguyen 2018 | SCR | 77 | 75.3 ± 8.3 | 4.4 ± 1.5 | 3.4 ± 1.1 | 21%W, 79%A | 9%O, 48%D | 77 | TEE and/ CTA at 90, 180, 365 | 3.9 | N/S | 2.6 | 2.6 |
| Osmancik 2020 | MCR | 201 | 73.4 ± 6.7 | 4.7 ± 1.5 | 3.1 ± 0.9 | 39%W, 61%A | 18%O, 82%D | 181 | TEE at 90 | 3.4 | 6(90 day) | 4.8 | 4.8 |
| Park 2009 | SCR | 73 | 72.7 ± 9.7 | N/R | N/R | PLAATO | 100%D | 52 | TEE at 90, 180 | 0 | - | 0 | 0 |
| Park 2018 | MCR | 60 | 74.4 ± 9.0 | 4.0 ± 1.6 | 3.2 ± 1.3 | Lambre | 100%D | 58 | TEE at 30, 180, 365 | 0 | - | 0 | 1.6 |
| Plicht 2013 | SCR | 34 | 74.0 ± 7.8 | 5.5 ± 1.4 | 4.2 ± 1.1 | A | 100%D | 30 | TEE at 30, 90, 180, 365 | 10 | 3(90 day) | 0 | 0 |
| Pracoń 2018 | SCR | 99 | 74 (68-80) | 4 (3-5) | 2 (1-3) | 41%W, 59%A | 100%D | 99 | CT/TEE at 45, 180, 365 | 5.1 | 2(180), 3(365 day) | 1 | 1 |
| Pracoń 2022 | SCR | 156 | 74 (68-81) | 4 (3-5) | 3 (2-3) | 44%W, 56%A | 100%D | 195 | CT/TEE at 45, 90, 180, 365 | 11.3 | N/S | 0.5 | 1.5 |
| Reddy 2013 | MCR | 150 | 72.5 ± 7.4 | 4.4 ± 1.7 | N/R | W | 100%D | 142 | TEE at 90, 365 | 4.2 | 5(164), 1(341day) | 2 | 2 |
| Regueiro 2018 | MCR | 101 | 76 (69-80) | 4.8 ± 1.6 | 4.2 ± 0.9 | 15%W, 85%A | 0%O, 71%D | 79 | TEE at 45, 180 | 2.5 | 2(180 day) | 7.6 | 12.7 |
| Saw 2017 | MCR | 106 | 74.8 ± 7.7 | 4.3 ± 1.5 | 3.2 ± 1.2 | W | 20%O, 73%D | 104 | TEE at 180 | 1 | 1(180 day) | 0 | 1.9 |
| Saw 2017 | MCR | 339 | 74.4 ± 7.5 | 4.3 ± 1.5 | 3.0 ± 1.2 | A | 6%O, 62%D | 339 | TEE at 134(88-227) | 3.2 | N/S | 0.9 | 2.1 |
| Sick 2007 | MCR | 75 | 68(47-83) | N/R | N/R | W | 100%O | 60 | TEE at 45, 180 | 6.7 | 4(180 day) | 0 | 2.7 |
| Szymala 2017 | SCR | 80 | 71.1 ± 8.9 | 4 ± 2 | 3.2 ± 0.8 | A | 0%O, 0%D | 77 | TEE at 90, 180 | 6.8 | 5(180 day) | 0 | 1.2 |
| Tung K 2016 | SCR | 47 | 74.9 ± 8.9 | 4.5 ± 1.4 | 3.0 ± 1.0 | W | 17%O, 23%D | 47 | N/S | 0 | - | 4.3 | 4.3 |
| Urena 2013 | MCR | 52 | 74 ± 8 | 5 (4–6) | 4 (3–4) | A | 4%O, 29%D | 52 | TEE at 180 | 0 | - | 1.9 | 3.8 |
| Wang G 2020 | SCR | 56 | 66.6 ± 8.4 | 3.7 ± 1.3 | 2.3 ± 0.9 | Lambre | 100%D | 56 | TEE at 90 | 3.6 | 2(90day) | 3.6 | 3.6 |
| Wang J 2021 | SCR | 97 | 65.4 ± 10.2 | 3.8 ± 1.5 | 2.5 ± 1.0 | 52%W, 48%Lambre | 100%O | 97 | TEE at 90 | 0 | - | 2.1 | 2.1 |
| Wiebe 2015 | SCR | 102 | 71.6 ± 8.8 | 4.3 ± 1.7 | N/R | W | 58%O, 42%D | 98 | TEE at 90, 180, 365 | 2 | 1(90), 1(180 day) | 2 | 1 |
| Wintgens 2018 | MCR | 349 | 63.1 ± 8.2 | 3(2-4) | 3(2-3) | W | 12%O, 44%D | 272 | TEE at 90 | 1.1 | 3(90 day) | 0.9 | 1.7 |
| Wolfrum 2016 | SCR | 169 | 73.1 ± 10.4 | 4.2 ± 1.7 | 2.9 ± 1.1 | A | 11%O, 89%D | 169 | TEE at 30, 180 | 7 | 12(180 day) | 0.6 | 0.6 |
| Yu J 2019 | SCR | 351 | 76.0 ± 8.0 | 3.9 ± 1.5 | 3.5 ± 1.1 | 98%W, 2%A | N/R | 351 | TEE at 45, 180 | 4 | N/S | N/S | 3.3 |
| Zhang X 2021 | SCR | 192 | 69.2 ± 8.1 | 2.8 ± 1.7 | 2.8 ± 1.0 | W | 100%O | 192 | TEE at 90, 180, 365 | 2.6 | 5(180day) | 2.6 | 3.1 |

N = number; SCR = single-center registry; MCR = multicenter registry; RCT = Randomized Clinical Trail; N/S = Not Specified; N/R = Not Reported; LAAC = left atrial appendage closure; W = WATCHMAN/ WATCHMAN FLX; A = Amplatzer (ACP or Amulet); OAC = oral anticoagulation; DAPT = dual antiplatelet; F/u = Follow-up; LAA = Left Atrial Appendage; TTE = trans-thoracic echocardiogram; TEE = transesophageal echocardiography; TOE = Transesophageal echocardiography; CTA = Computed Tomography Angiography; CAR = continuous access registry; DRT = device related thrombus; Dx = Diagnosis; SE = systemic embolism.

**Supplementary Table 2.** Comparisons of the incidence of DRT and related ischemic events among single-seal (SS), dual-seal (DS) and other devices.

**A**

|  | **SS** | | **DS** | | **Other Devices** | |
| --- | --- | --- | --- | --- | --- | --- |
|  | Number | % | Number | % | Number | % |
| Total patient No | 6,446 |  | 3,101 |  | 89 |  |
| No of patients at follow-up | 6,190 | 96 | 3,023 | 97 | 85 | 96 |
| DRT incidence | 171/6,190 | 2.8 | 78/3,023 | 3.6 | 4/85 | 4.7 |
| DRT associated stroke | 26/171 | 16 | 8/78 | 10 | 0/4 | 0 |
| Event incidence not reported | 14/171 | 8 | 54/78 | 69 | 4/4 | 100 |
| No event associated with DRT | 131/171 | 76 | 16/78 | 21 | 0/4 | 0 |

**B**

|  | **SS** | | **DS** | | **Other Devices** | |
| --- | --- | --- | --- | --- | --- | --- |
|  | Number | % | Number | % | Number | % |
| Total patient No | 6,446 |  | 3,101 |  | 89 |  |
| No of patients at follow-up | 6,190 | 96 | 3,023 | 97 | 85 | 96 |
| DRT incidence | 171/6,190 | 2.8 | 78/3,023 | 2.6 | 4/85 | 4.7 |
| DRT associated systemic embolism | 32/171 | 19 | 3/78 | 4 | 0/4 | 0 |
| Event incidence not reported | 26/171 | 15 | 38/78 | 49 | 4/4 | 100 |
| No event associated with DRT | 113/171 | 66 | 37/78 | 47 | 0/4 | 0 |

(**A**) A comparison of the incidence of device related thrombus (DRT) and related stroke between SS and DS devices. (**B**) A comparison of the incidence of DRT and related systemic embolism between SS and DS devices.

**Supplementary Table 3.** Comparison of the incidence of DRT and related ischemic events among different type of devices.

**A**

|  | **WATCHMAN** | | **AMPLATZER** | | **Lariat** | | **Occlutech** | | **Lambre** | | **PLAATO** | |
| --- | --- | --- | --- | --- | --- | --- | --- | --- | --- | --- | --- | --- |
|  | Number | % | Number | % | Number |  | Number |  | Number |  | Number |  |
| Total patient No | 6,343 |  | 2,832 |  | 89 |  | 30 |  | 269 |  | 73 |  |
| No of patients at follow-up | 6,110 | 96 | 2,788 | 98 | 85 | 96 | 28 | 93 | 235 | 87 | 52 | 71 |
| DRT incidence | 169/6,110 | 2.8 | 74/2,788 | 2.7 | 4/85 | 4.7 | 0/28 | 0 | 4/235 |  | 0/52 | 0 |
| DRT associated stroke | 26/169 |  | 2/74 |  | 0/4 | 0 | - | - | 0/4 | 0 | - | - |
| Event incidence not reported | 23/169 |  | 41/74 |  | 4/4 | 100 | - | - | 2/4 | 50 | - | - |
| No event associated with DRT | 120/169 |  | 31/74 |  | 0/4 | 0 | - | - | 2/4 | 50 | - | - |

**B**

|  | **WATCHMAN** | | **AMPLATZER** | | **Lariat** | | **Occlutech** | | **Lambre** | | **PLAATO** | |
| --- | --- | --- | --- | --- | --- | --- | --- | --- | --- | --- | --- | --- |
|  | Number | % | Number | % | Number |  | Number |  | Number |  | Number |  |
| Total patient No | 6,343 |  | 2,832 |  | 89 |  | 30 |  | 269 |  | 73 |  |
| No of patients at follow-up | 6,110 | 96 | 2,788 | 98 | 85 | 96 | 28 | 93 | 235 | 87 | 52 | 71 |
| DRT incidence | 169/6,110 | 2.8 | 74/2,788 | 2.7 | 4/85 | 4.7 | 0/28 | 0 | 4/235 |  | 0/52 | 0 |
| DRT associated systemic embolism | 34/169 | 20 | 3/74 | 4 | 0/4 | 0 | - | - | 0/4 | 0 | - | - |
| Event incidence not reported | 22/169 | 13 | 36/74 | 49 | 4/4 | 100 | - | - | 2/4 | 50 | - | - |
| No event associated with DRT | 113/169 | 67 | 35/74 | 47 | 0/4 | 0 | - | - | 2/4 | 50 | - | - |

(**A**) A comparison of the incidence of DRT and related stroke among different type of devices. (**B**) A comparison of the incidence of DRT and related systemic embolism between different devices.

**Supplementary Table 4.** Methodological quality assessment of 59 studies.

**A**

| **Study name/**  **Publication year** | **①** | **②** | **③** | **④** | **⑤** | **⑥** | **⑦** | **⑧** | **Total** |
| --- | --- | --- | --- | --- | --- | --- | --- | --- | --- |
| Abelson 2021 | 1 | 2 | 1 | 2 | 0 | 2 | 1 | 0 | 9 |
| Aminian 2019 | 2 | 2 | 2 | 2 | 0 | 1 | 2 | 0 | 11 |
| Bartus 2013 | 1 | 2 | 2 | 2 | 1 | 1 | 2 | 0 | 11 |
| Bellmann 2016 | 2 | 2 | 2 | 1 | 0 | 1 | 2 | 0 | 10 |
| Bertrand 2018 | 2 | 2 | 2 | 1 | 0 | 2 | 2 | 0 | 11 |
| Boersma 2017 | 2 | 2 | 2 | 2 | 0 | 2 | 1 | 0 | 11 |
| Chen M 2020 | 2 | 2 | 2 | 1 | 0 | 1 | 2 | 0 | 10 |
| Chiu F 2021 | 2 | 2 | 2 | 2 | 0 | 2 | 2 | 0 | 12 |
| Chun K 2013 | 2 | 2 | 2 | 2 | 0 | 2 | 2 | 0 | 12 |
| Cochet 2018 | 2 | 2 | 2 | 1 | 0 | 2 | 2 | 0 | 11 |
| Darmon 2020 | 2 | 2 | 2 | 2 | 0 | 2 | 2 | 0 | 12 |
| De Backer 2014 | 1 | 2 | 2 | 1 | 0 | 1 | 2 | 0 | 10 |
| Della 2021 | 2 | 2 | 2 | 2 | 0 | 2 | 2 | 0 | 12 |
| Du X 2019 | 2 | 2 | 2 | 2 | 0 | 1 | 2 | 0 | 11 |
| Dukkipati 2018 | 2 | 2 | 2 | 2 | 0 | 2 | 2 | 0 | 12 |
| Enomoto 2016 | 2 | 2 | 2 | 1 | 0 | 1 | 2 | 0 | 10 |
| Fan Y 2019 | 2 | 2 | 2 | 2 | 0 | 1 | 2 | 0 | 11 |
| Faroux 2021 | 2 | 2 | 2 | 2 | 0 | 2 | 2 | 0 | 12 |
| Fassini 2016 | 2 | 2 | 1 | 2 | 0 | 2 | 2 | 0 | 11 |
| Fukunaga 2022 | 1 | 1 | 2 | 2 | 0 | 1 | 2 | 0 | 9 |
| Gu ZC 2020 | 2 | 2 | 2 | 1 | 0 | 1 | 2 | 0 | 10 |
| Guerios 2017 | 2 | 2 | 1 | 1 | 0 | 2 | 2 | 0 | 10 |
| Häner 2021 | 2 | 2 | 2 | 2 | 0 | 2 | 2 | 0 | 12 |
| Huang H 2017 | 2 | 2 | 2 | 2 | 2 | 2 | 2 | 0 | 14 |
| Huang W 2017 | 2 | 2 | 2 | 1 | 0 | 0 | 2 | 0 | 9 |
| Jalal 2017 | 2 | 1 | 2 | 2 | 0 | 2 | 2 | 0 | 11 |
| Kebernik 2015 | 2 | 2 | 2 | 1 | 0 | 0 | 0 | 0 | 7 |
| Kita 2020 | 2 | 2 | 2 | 2 | 0 | 2 | 2 | 0 | 12 |
| Kubo 2017 | 2 | 2 | 2 | 1 | 0 | 2 | 2 | 0 | 11 |
| Kuroki 2020 | 1 | 1 | 2 | 2 | 0 | 2 | 2 | 0 | 10 |
| Ledwoch 2016 | 2 | 2 | 2 | 2 | 0 | 2 | 1 | 0 | 11 |
| Li W 2022 | 2 | 2 | 2 | 1 | 0 | 2 | 0 | 0 | 9 |
| Li X 2021 | 2 | 1 | 2 | 1 | 0 | 0 | 2 | 0 | 8 |
| Matsuo 2014 | 2 | 2 | 2 | 1 | 0 | 1 | 0 | 0 | 8 |
| Mazzone 2018 | 2 | 2 | 2 | 2 | 0 | 2 | 2 | 0 | 12 |
| Merella 2019 | 2 | 0 | 2 | 1 | 0 | 2 | 0 | 0 | 7 |
| Mo B 2021 | 2 | 2 | 2 | 2 | 2 | 2 | 2 | 0 | 14 |
| Nguyen 2018 | 2 | 2 | 2 | 2 | 0 | 2 | 2 | 0 | 12 |
| Park 2009 | 2 | 2 | 2 | 1 | 0 | 2 | 2 | 0 | 11 |
| Park 2018 | 2 | 2 | 1 | 2 | 0 | 2 | 0 | 0 | 9 |
| Plicht 2013 | 2 | 2 | 2 | 1 | 0 | 2 | 1 | 0 | 10 |
| Pracoń 2018 | 2 | 2 | 2 | 2 | 0 | 2 | 2 | 0 | 12 |
| Pracoń 2022 | 2 | 2 | 2 | 2 | 0 | 2 | 1 | 0 | 11 |
| Reddy 2013 | 2 | 2 | 2 | 2 | 0 | 2 | 2 | 0 | 12 |
| Regueiro 2018 | 2 | 2 | 2 | 1 | 0 | 2 | 2 | 0 | 11 |
| Saw 2017 | 2 | 2 | 2 | 2 | 0 | 1 | 2 | 0 | 11 |
| Saw 2017 | 2 | 2 | 2 | 2 | 0 | 2 | 2 | 0 | 12 |
| Sick 2007 | 2 | 2 | 2 | 2 | 2 | 1 | 2 | 0 | 13 |
| Szymala 2017 | 2 | 2 | 2 | 1 | 0 | 0 | 2 | 0 | 9 |
| Tung K 2016 | 2 | 1 | 2 | 2 | 0 | 1 | 2 | 0 | 10 |
| Urena 2013 | 2 | 2 | 2 | 2 | 0 | 2 | 2 | 0 | 12 |
| Wang G 2020 | 2 | 2 | 1 | 1 | 0 | 2 | 2 | 0 | 10 |
| Wang J 2021 | 2 | 2 | 2 | 2 | 0 | 2 | 2 | 0 | 12 |
| Wiebe 2015 | 2 | 2 | 2 | 2 | 0 | 2 | 2 | 0 | 12 |
| Wintgens 2018 | 2 | 1 | 2 | 2 | 0 | 2 | 2 | 0 | 11 |
| Wolfrum 2016 | 1 | 1 | 2 | 2 | 0 | 2 | 1 | 0 | 9 |
| Yu J 2019 | 2 | 2 | 2 | 1 | 0 | 2 | 1 | 0 | 10 |
| Zhang X 2021 | 2 | 2 | 2 | 1 | 0 | 1 | 2 | 0 | 10 |

**B**

| **Study name/**  **Publication year** | **Random Sequence generation** | **Allocation concealment** | **Blinding of participants and personnel** | **Blinding of outcome assessment** | **Incomplete outcome data** | **Selective outcome reporting** | **Other bias** |
| --- | --- | --- | --- | --- | --- | --- | --- |
| Osmancik 2020 | Low risk | Low risk | Low risk | Low risk | Low risk | Low risk | Low risk |

(**A**) Observational studies assessed by the Methodological items for non-randomized studies (MINORS). The MINORS includes 8 items: ①A clearly stated aim; ②Inclusion of consecutive patients; ③Prospective collection of data; ④Endpoints appropriate to the aim of the study; ⑤Unbiased assessment of the study endpoint; ⑥Follow-up period appropriate to the aim of the study; ⑦Loss to follow up less than 5%; ⑧Prospective calculation of the study size. (**B**) RCTs assessed by the Cochrane Collaboration's tool.


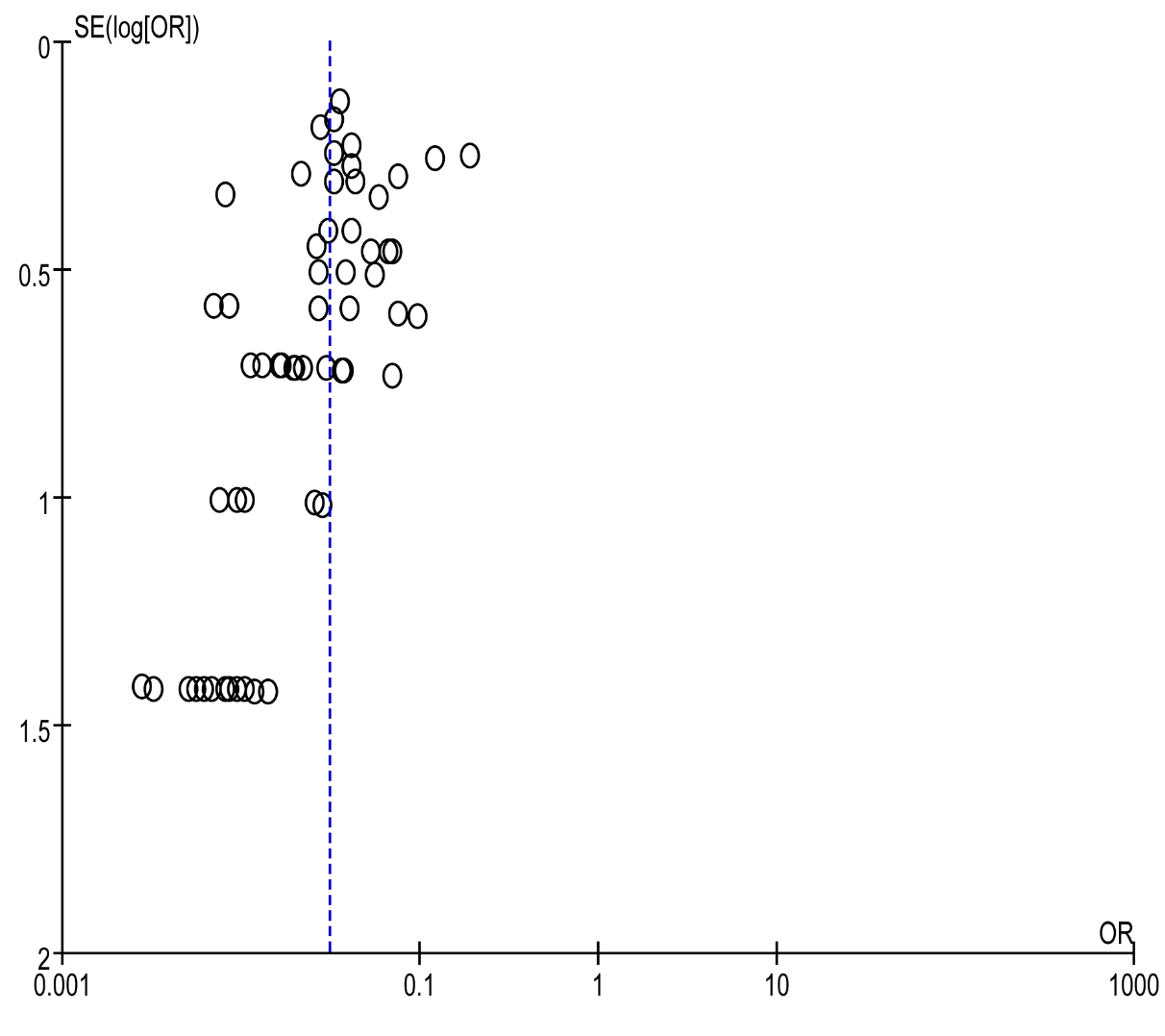


**Supplementary Figure 1.** Funnel plot for publication bias in eligible studies.


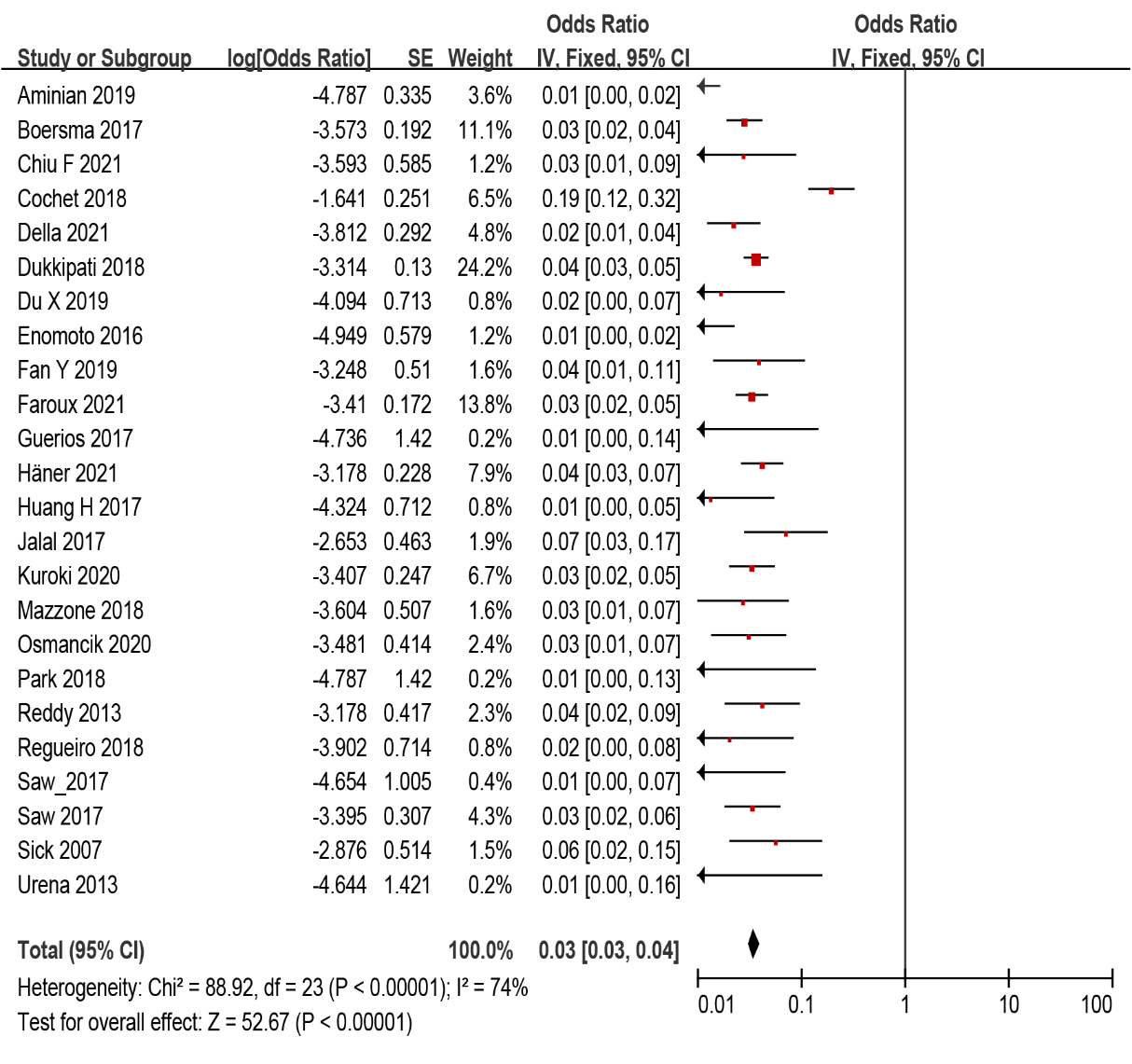


**Supplementary Figure 2.** The incidence of DRT after LAAC in multicenter registries (MCR) and RCT.


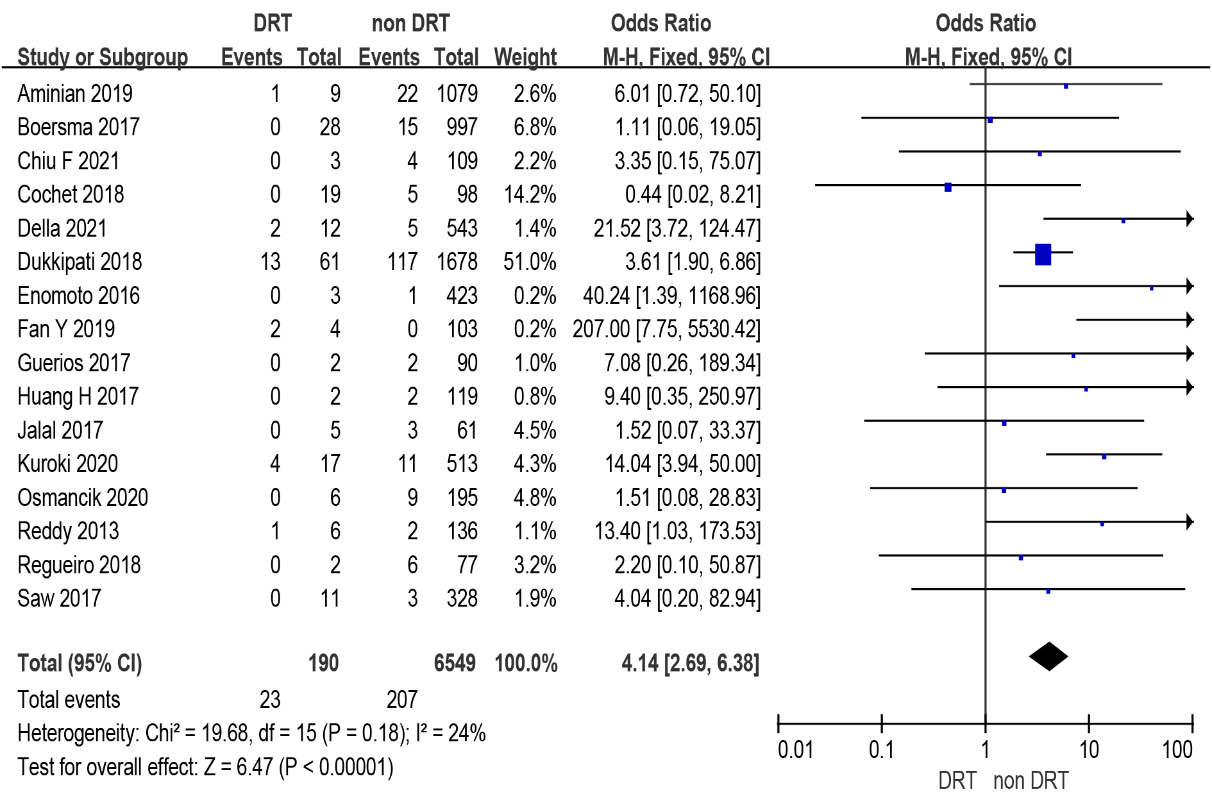


**Supplementary Figure 3.** Forest plot of stroke in DRT patients and non-DRT patients: MCR and RCT.


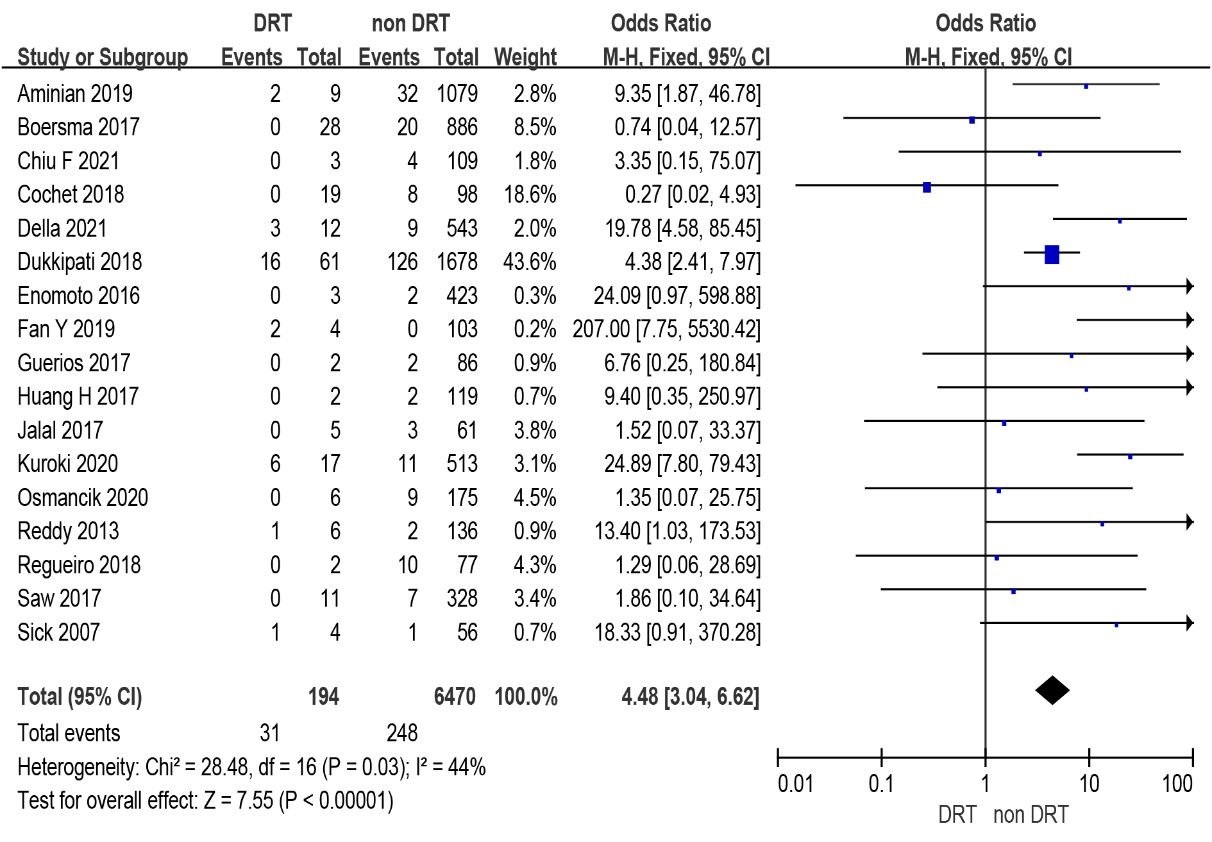


**Supplementary Figure 4.** Forest plot of systemic embolism (SE) in DRT patients and non-DRT patients: MCR and RCT.


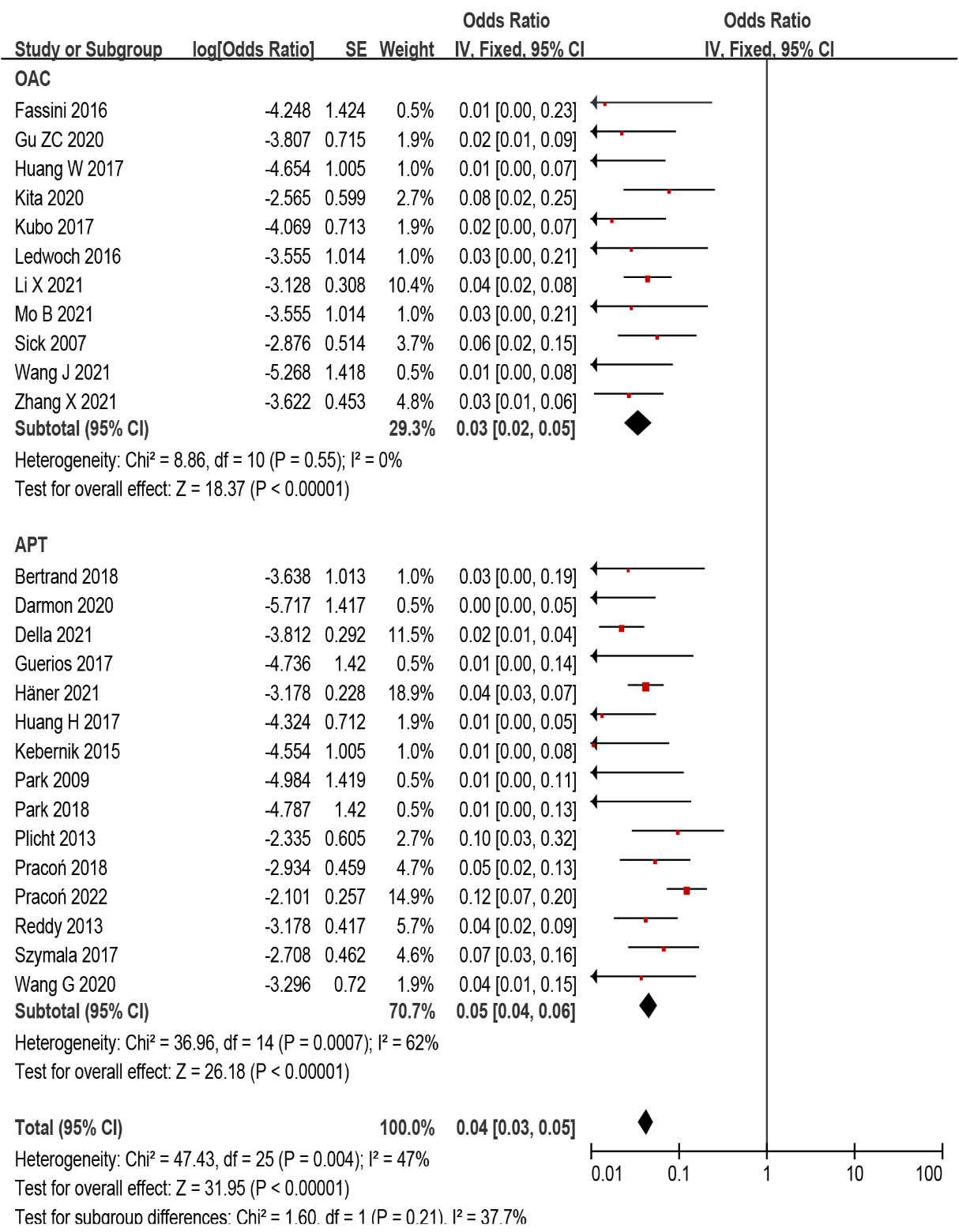


**Supplementary Figure 5.** Forest plot of incidence of DRT in patients with subgroup analysis based on anti-thrombotic therapy.
